# Magnitude and kinetics of multifunctional CD4^+^ and CD8β^+^ T cells in pigs infected with swine influenza A virus

**DOI:** 10.1186/s13567-015-0182-3

**Published:** 2015-05-14

**Authors:** Stephanie C Talker, Hanna C Koinig, Maria Stadler, Robert Graage, Eva Klingler, Andrea Ladinig, Kerstin H Mair, Sabine E Hammer, Herbert Weissenböck, Ralf Dürrwald, Mathias Ritzmann, Armin Saalmüller, Wilhelm Gerner

**Affiliations:** Institute of Immunology, Department of Pathobiology, University of Veterinary Medicine, Vienna, Austria; University Clinic for Swine, Department for Farm Animals and Veterinary Public Health, University of Veterinary Medicine, Vienna, Austria; Institute of Pathology and Forensic Veterinary Medicine, Department of Pathobiology, University of Veterinary Medicine, Vienna, Austria; Viral Vaccines, Business Unit Animal Health, IDT Biologika GmbH, Dessau-Rosslau, Germany; Present address: Institute of Veterinary Pathology, Vetsuisse-Faculty, University of Zurich, Zurich, Switzerland; Present address: Clinic for Swine, Ludwig-Maximilians-University, Munich, Germany

## Abstract

Although swine are natural hosts for influenza A viruses, the porcine T-cell response to swine influenza A virus (FLUAVsw) infection has been poorly characterized so far. We have studied Ki-67 expression and FLUAVsw-specific production of IFN-γ, TNF-α and IL-2 in CD4^+^ and CD8β^+^ T cells isolated from piglets that had been intratracheally infected with a H1N2 FLUAVsw isolate. IFN-γ^+^TNF-α^+^IL-2^+^ multifunctional CD4^+^ T cells were present in the blood of all infected animals at one or two weeks after primary infection and their frequency increased in four out of six animals after homologous secondary infection. These cells produced higher amounts of IFN-γ, TNF-α and IL-2 than did CD4^+^ T cells that only produced a single cytokine. The vast majority of cytokine-producing CD4^+^ T cells expressed CD8α, a marker associated with activation and memory formation in porcine CD4^+^ T cells. Analysis of CD27 expression suggested that FLUAVsw-specific CD4^+^ T cells included both central memory and effector memory populations. Three out of six animals showed a strong increase of Ki-67^+^perforin^+^ CD8β^+^ T cells in blood one week post infection. Blood-derived FLUAVsw-specific CD8β^+^ T cells could be identified after an in vitro expansion phase and were multifunctional in terms of CD107a expression and co-production of IFN-γ and TNF-α. These data show that multifunctional T cells are generated in response to FLUAVsw infection of pigs, supporting the idea that T cells contribute to the efficient control of infection.

**Electronic supplementary material:**

The online version of this article (doi:10.1186/s13567-015-0182-3) contains supplementary material, which is available to authorized users.

## Introduction

Pigs are natural hosts for influenza A viruses and infections of humans with swine influenza A viruses (FLUAVsw) have been reported [1]. Moreover, the pig is considered as a “mixing vessel” i.e. a species where reassortments between avian and mammalian influenza virus strains can occur which may lead to the emergence of novel pandemic strains in humans. For example, in the 2009 pandemic H1N1 virus, genes closely related to swine North American and Eurasian H1N1 viruses were identified [2]. The 2009 pandemic H1N1 virus was frequently transmitted from farmers to pigs during the last years, thereby reflecting the zoonotic potential of this virus. As a consequence, this transmission established a new lineage of pandemic viruses (pandemic H1N2) in pigs via reassortment with circulating swine influenza viruses [3].

These observations, but also economic and animal welfare issues of FLUAVsw infections in pig production units, justify investigations on pig-FLUAVsw host-pathogen interactions. Of note, FLUAVsw infections are usually rapidly controlled by the porcine immune system and an elimination of replicating virus from the respiratory tract within one week has been reported [4]. Neutralizing antibodies appear in serum within seven days post inoculation [4]. It is assumed that these antibodies play a major role in control of infection, although a production of IgA antibodies by B cells in the nasal mucosa has also been reported [5].

The rapid control of FLUAVsw infections suggests that also cell-mediated immune responses contribute to viral clearance. However, while abundant knowledge exists on the role of influenza virus-specific CD4^+^ and CD8^+^ T cells in mice and humans [6], their role has not been studied in great detail in pigs. A FLUAVsw-specific proliferation of lymphocytes isolated from blood has been reported following infection of pigs with H3N2 and H1N1 FLUAVsw strains [7-9]. One study demonstrated the proliferation of blood-derived CD4^+^ and CD8^+^ T cells following vaccination with a human pandemic H1N1 vaccine [10]. Also, the presence of H1N1-specific IFN-γ producing T cells in tracheobronchial lymph nodes, spleen and nasal mucosa has been described [5]. More recently, increased frequencies of cytolytic T cells (CTLs), CD4^+^CD8α^+^ T cells and regulatory T cells have been reported in lung tissue and bronchoalveolar lavage fluid of H1N1-infected pigs six days post infection [11].

However, none of these studies investigated the phenotype and functional properties of FLUAVsw-specific T cells in detail. Taking into account the rapid clearance of FLUAVsw infections, we hypothesized that highly differentiated CD4^+^ and CD8β^+^ T cells with multiple effector functions are involved in protective immune responses. Accordingly, we performed a detailed phenotypic and functional analysis of FLUAVsw-specific T cells occurring in blood of pigs experimentally infected with a FLUAVsw H1N2 isolate.

## Materials and methods

### Animals and virus

Nine three-week-old crossbred piglets ([Landrace × Large White] × Pietrain) were derived from a conventional breeding farm in Lower Austria and were kept in a biosafety level 2 facility at the University of Veterinary Medicine Vienna. Sows at the farm were free of FLUAVsw-specific antibodies. This was tested by a commercial ELISA (Ingezim Influenza A, 1.0.FLU.K3, Ingenasa, Madrid, Spain) at regular intervals. All piglets were vaccinated against *Mycoplasma hyopneumoniae* (MycoFLEX®, Boehringer Ingelheim, Ingelheim, Germany) and against porcine circovirus type 2 (CircoFLEX®, Boehringer Ingelheim) at three weeks of age. After arrival, the piglets were divided into a control group (three animals) and an infection group (six animals). Seronegativity for antibodies against Influenza A was confirmed by the Ingezim Influenza A, 1.0.FLU.K3 ELISA (Ingenasa) one day before FLUAVsw infection (four weeks of age). Piglets were infected twice at an interval of four weeks. Each time, 10 mL of virus suspension containing the FLUAVsw isolate A/swine/Kitzen/IDT6142/2007 (H1N2) with 10^7.25^ TCID_50_/mL was administered intratracheally by the use of a laryngoscope to anesthetized piglets (Narketan®: Ketaminhydrochlorid 10 mg/kg body weight (BW), Vetoquinol, Lure, France; Stresnil®: Azaperon 1.2 mg/kg BW, Janssen Pharmaceutical, Beerse, Belgium). Control animals underwent the same procedure, but received phosphate buffered saline (PBS) instead. Daily, clinical examinations were carried out and recorded in a clinical score. Additionally, rectal temperature and body weight were measured daily and weekly, respectively. Euthanasia of pigs was performed either two or five weeks following the second infection. Piglets were anesthetized (Narketan®, Stresnil®), before T61® (T61®: Embutramid, Mebezoniumiodid, Tetracainhydrochlorid, 1 mL/10 kg BW, MSD, Whitehouse Station, NJ, USA) was administered. At necropsy, gross lung lesions were documented and samples from all seven lung lobes were fixed in 10% neutral buffered formalin. The samples were processed for 3 μm thick paraffin sections and stained with hematoxylin and eosin. The slides were examined for presence and quantity of the following parameters: (1) septal infiltration with mononuclear cells, (2) lobular atelectasis and interstitial fibrosis with metaplasia of alveolar epithelium, (3) interstitial formation of lymphoid follicles and/or bronchus-associated lymphoid tissue (BALT) hyperplasia.

The animal experiment was approved by the institutional ethics committee, the Advisory Committee for Animal Experiments (§12 of Law for Animal Experiments, Tierversuchsgesetz – TVG) and the Federal Ministry for Science and Research (reference number BMWF-68.205/0180-II/3b/2011).

FLUAVsw/Kitzen/IDT6142/2007 (H1N2) was originally isolated from an influenza outbreak in a pig herd in Saxony, Germany. It was isolated on primary porcine thymus cells and thereafter passaged on embryonated hen eggs (three passages) and on MDBK cells. The forth passage on MDBK cells was used for the experiments. The virulence of this passage of this strain had been proved by intratracheal and aerosol infection trials in pigs at IDT Biologika GmbH during the development of a swine influenza vaccine (EMEA/V/C/153) prior to the experiments reported here. The strain is able to induce dyspnea and fever in pigs at infection doses of 10^8.55^ TCID_50_ MDBK at intratracheal infection. The sequences of hemagglutinin and neuraminidase are available from NCBI GenBank, accession numbers GQ161145 and GQ161146, respectively. This virus belongs to the human-like H1N2 swine influenza A viruses (huH1N2) which contain the hemagglutinin of H1N1 influenza A viruses which circulated in the human population in the 1970s/1980s and the neuraminidase of swine human-like H3N2 influenza A viruses isolated in the UK in the early 1990s [12].

### Sample collection and isolation of peripheral blood mononuclear cells (PBMCs)

Blood samples were taken weekly by puncturing the anterior *vena cava* or the jugular vein. Leukograms (total white blood cell counts and differentials) were generated by using the hematology system ADVIA2120i (Siemens Healthcare Diagnostics, Eschborn, Germany). Serum samples were frozen at −20 °C for subsequent detection of neutralizing antibodies. PBMCs were isolated from heparinized blood by density gradient centrifugation with lymphocyte separation medium (PAA Laboratories, Pasching, Austria) as described elsewhere [13]. Isolated PBMCs were either directly used for ex vivo multicolor flow cytometry (FCM) and IFN-γ ELISpot assays or were cryopreserved for later in vitro experiments.

### Serum neutralization test

Neutralizing antibodies were analyzed via serum neutralization tests as previously described [12]. Briefly, a serial dilution of sera was prepared and mixed with one of four different virus strains: A/sw/Bakum/1832/2000 (huH1N2), A/sw/Haselünne/IDT2617/2003 (avH1N1), A/sw/Bakum/IDT1769/2003 (huH3N2) and A/Jena/VI5256/2009 (panH1N1). Each strain was adjusted to 100 TCID_50_. After incubation for 1 h at 37 °C, the virus-incubated sera were added to MDBK cell monolayers in microtiterplates. The medium of MDBK cell cultures was supplemented with porcine trypsin (γ-irradiated, final concentration 4 BAEE units/mL medium; Sigma-Aldrich, Schnelldorf, Germany) at the time of infection and again after 24 h. After 48 h of incubation at 37 °C, cells were fixed with acetone and investigated by indirect immunofluorescence, followed by ND_50_ calculation.

### Phenotypic analysis of PBMCs performed ex vivo

For phenotypic analysis of CD4^+^ and CD8β^+^ T cells, freshly isolated PBMCs were suspended in PBS (without Ca^2+^/Mg^2+^, PAA) and 10% (v/v) porcine plasma (in-house preparation) and adjusted to 1 × 10^6^ cells per sample. Monoclonal antibodies (mAbs) and secondary reagents used for cell surface staining are listed in Table 1. For detection of intracellular perforin and Ki-67, cells were fixed and permeabilized by in-house prepared fixation and permeabilization buffers containing saponin, as described previously [14]. Staining was performed in 96-well microtiterplates and cells were incubated for 20 min at 4 °C in the fridge. Free binding sites of secondary antibodies were blocked with whole mouse IgG molecules (2 μg per sample, Jackson ImmunoResearch, West Grove, PA, USA) during an additional incubation step prior to fixation and permeabilization.

**Table 1:** **Antibody panels**

**Antigen**	**Clone**	**Isotype**	**Fluorochrome**	**Labeling strategy**	**Source of primary Ab**
*Ex vivo Ki-67/CD4* ^*+*^ *T cells*					
CD3	PPT3	IgG1	PE	secondary antibody^a^	in house
CD4	74-12-4	IgG2b	Alexa488	secondary antibody^b^	in house
CD8α	11/295/33	IgG2a	PE-Cy7	secondary antibody^c^	in house
CD27	b30c7	IgG1	Alexa647	directly conjugated^d^	in house
CD45RC	3a56	IgG1	PerCP-Cy5.5	directly conjugated^e^	in house
SLA-DR	MSA3	IgG2a	Qdot605	directly conjugated	in house^f^
Ki-67	B56	IgG1	V450	directly conjugated	BD Biosciences
*Ex vivo Ki-67/CD8β* ^*+*^ *T cells*					
CD3	PPT3	IgG1	PE	secondary antibody^a^	in house
CD8β	PG164A	IgG2a	Alexa488	Zenon labeling kit^g^	VMRD
CD27	b30c7	IgG1	Alexa647	directly conjugated^d^	in house
SLA-DR	MSA3	IgG2a	Qdot605	directly conjugated	in house^f^
Perforin	δ-G9	IgG2b	PerCP-eFluor710	directly conjugated	eBioscience
Ki-67	B56	IgG1	V450	directly conjugated	BD Biosciences
*Triple cytokine staining/CD4* ^*+*^ *T cells*					
CD4	74-12-4	IgG2b	Alexa488	secondary antibody^b^	in house
CD8α	11/295/33	IgG2a	PE-Cy7	secondary antibody^c^	in house
CD27	b30c7	IgG1	BV421	biotin-streptavidin^h^	in house
IFN-γ	P2G10	IgG1	PE	directly conjugated	BD Biosciences
TNF-α	MAb11	IgG1	BV605	directly conjugated	BioLegend
IL-2	A150D3F1	IgG2a	APC	directly conjugated^i^	Life Technologies
*Triple cytokine staining/CD8β* ^*+*^ *T cells*					
CD3	BB23-8E6-8C8	IgG2a	PE-Cy7	directly conjugated	BD Biosciences
CD8β	PG164A	IgG2a	Alexa488	Zenon labeling kit^g^	VMRD
CD27	b30c7	IgG1	BV421	biotin-streptavidin^h^	in house
IFN-γ	P2G10	IgG1	PE	directly conjugated	BD Biosciences
TNF-α	MAb11	IgG1	BV605	directly conjugated	BioLegend
IL-2	A150D3F1	IgG2a	APC	directly conjugated^i^	Life Technologies
*In vitro expanded CD8β* ^*+*^ *T cells*					
CD4	74-12-4	IgG2b	PerCP-Cy5.5	directly conjugated	BD Biosciences
CD8β	PG164A	IgG2a	Alexa488	secondary antibody^j^	VMRD
CD107a	4E9/11	IgG1	Alexa647	directly conjugated	AbD Serotec
Pan-γδ	PPT16	IgG2b	PE-Cy7	biotin-streptavidin^k^	in house
IFN-γ	P2G10	IgG1	PE	directly conjugated	BD Biosciences
TNF-α	MAb11	IgG1	BV605	directly conjugated	BioLegend

^a^Goat anti-Mouse IgG1-PE, Southern Biotech.

^b^Goat anti-Mouse IgG2b-Alexa488, Life Technologies.

^c^Goat anti-Mouse IgG2a-PE-Cy7, Southern Biotech.

^d^Alexa Fluor-647 Protein Labeling Kit, Life Technologies.

^e^Lightning-Link™ PerCP-Cy5.5 Tandem Conjugation Kit, Innova Biosciences.

^f^Custom conjugation by Life Technologies.

^g^IgG2a-Alexa488 Zenon labeling kit, Life Technologies.

^h^Streptavidin-BV421, BioLegend.

^i^Lightning-Link™ APC Conjugation Kit, Innova Biosciences.

^j^Goat anti-Mouse IgG2a-Alexa488, Life Technologies.

^k^Streptavidin-PE-Cy7, eBioscience.

### ELISpot assays for IFN-γ production

96-well MultiScreen IP plates (Millipore, Billerica, MA, USA) were coated with mouse anti-swine IFN-γ mAb (clone pIFN-γ, Mabtech, Nacka Strand, Sweden) overnight at 4 °C (100 μL/well; 10 μg/mL in PBS), washed with PBS and subsequently blocked with cell culture medium (RPMI 1640 with stable glutamine supplemented with 10% [v/v] fetal calf serum, 100 IU/mL penicillin and 0.1 mg/mL streptomycin, all from PAA) for one hour at 37 °C. Per well, 3 × 10^5^ freshly isolated PBMCs were incubated with the FLUAVsw infection strain (multiplicity of infection, MOI 0.1) or mock for 24 h at 37 °C. Samples incubated in cell culture medium served as additional negative control. Thereafter, plates were washed and incubated with biotin-labeled mouse anti-IFN-γ mAbs (clone PAN, Mabtech, 100 μL/well; 0.5 μg/mL in PBS) for one hour at room temperature. This was followed by incubation with streptavidin-alkaline phosphatase (1:2000 in PBS supplemented with 0.01% Tween20 and 0.1% BSA, Roche, Mannheim, Germany) for one hour at room temperature and subsequent addition of 5-bromo-4-chloro-3-indolyl phosphate/nitro blue tetrazolium substrate (100 μL/well, Sigma-Aldrich) according to manufacturer’s instructions. After intense washing and drying of plates, spots were analyzed with an AID ELISpot reader (AID, Straßberg, Germany).

### Intracellular cytokine staining

For intracellular staining of IFN-γ, TNF-α and IL-2 in CD4^+^ and CD8β^+^ T cells, PBMCs were defrosted and rested at 37 °C for eight hours (5 × 10^5^ cells per well in 150 μL cell culture medium). Thereafter, FLUAVsw (infection strain, MOI = 0.1) was added to microcultures and left overnight for 18 h at 37 °C in a total volume of 200 μL. During the last four hours, Brefeldin A (BD GolgiPlug™, BD Biosciences, San Jose, CA, USA) was present in microcultures at a final concentration of 1 μg/mL. Mock- and medium-incubated cultures served as controls. For subsequent FCM staining in microtiterplates, cells were washed in PBS (without Ca^2+^/Mg^2+^) containing 3% (v/v) fetal calf serum and incubated with antibodies and secondary reagents listed in Table 1. Free binding sites of secondary antibodies were blocked with whole mouse IgG molecules (details see above) and Near-IR LIVE/DEAD stain kit (Life Technologies, Carlsbad, CA, USA) was used according to manufacturer’s instructions. For fixation and permeabilization of cells, BD Cytofix/Cytoperm and BD Perm/Wash (both BD Biosciences) were used according to manufacturer’s instructions.

### In vitro expansion and functional analysis of CD8β^+^ T cells

Defrosted PBMCs were labeled with CellTrace™ Violet Cell Proliferation Kit (Life Technologies) as described elsewhere [15]. Per well, 5 × 10^5^ violet-stained PBMCs were plated together with FLUAVsw (infection strain, MOI 0.1) and were incubated at 37 °C for five days. At the fifth day, cells were restimulated for a second time with FLUAVsw (MOI 0.1) for another 18 h. Four hours prior to harvesting of cells, anti-CD107a mAb was added, together with Brefeldin A (GolgiPlug™, BD Biosciences; final concentration 1 μg/mL) and Monensin (GolgiStop™, BD Biosciences; final concentration 1 μg/mL). Cell surface labeling and intracellular cytokine staining was performed with reagents listed in Table 1, under the conditions described above.

### FCM analysis

FCM measurements were performed using a FACSCanto™ II flow cytometer (BD Biosciences) equipped with three lasers (405, 488 and 633 nm) and a High Throughput Sampler. Compensation was calculated automatically with single-stain samples. For analysis of ex vivo Ki-67 expression, at least 1 × 10^5^ lymphocytes were collected. Between 5 × 10^5^ and 1 × 10^6^ lymphocytes were recorded for detection of intracellular cytokines. Gating strategies for identification of lymphocytes, doublet discrimination and exclusion of dead cells are shown in Additional file 1. Data was processed by FACSDiva software (Version 6.1.3; BD Biosciences) or FlowJo software (Version 7.6.5; Tree Star, Ashland, OR, USA) and transferred to Microsoft Excel (Office 2010; Microsoft, Redmond, WA, USA) for further calculations and preparation of graphs.

### Swine leukocyte antigen haplotyping

Pigs were genotyped for their swine leukocyte antigen (SLA) class I and II haplotypes by running low-resolution PCR screening assays (PCR-SSP) on PBMC-derived genomic DNA as previously described [16].

## Results

### Clinical signs and pathological findings following FLUAVsw infection

**Figure 1:**
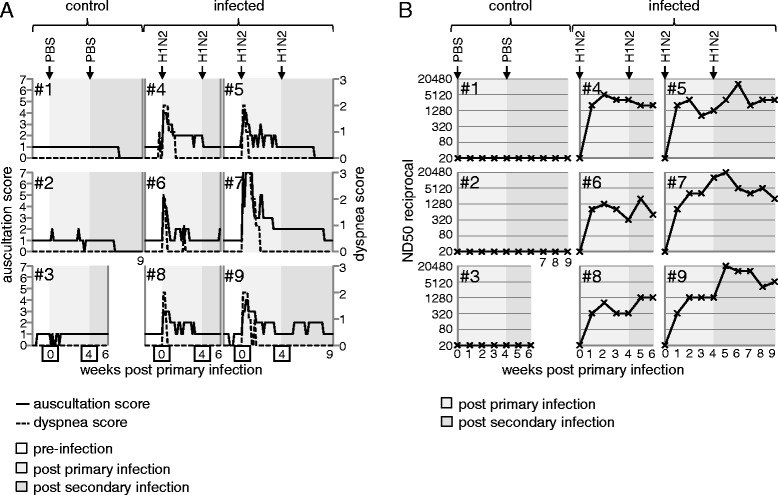
**Clinical score and H1N2-neutralizing antibody titers.** **(A)** From one week prior to the first infection until the end of the study either six or nine weeks post primary infection, piglets were daily monitored for clinical signs of disease. Auscultation score (solid line; 0 = physiologic, 1 = low-grade intensified vesicular, 2 = middle-grade intensified vesicular, 3 = high-grade intensified vesicular, 4 = bronchial, 5 = wheezing, 6 = rales, 7 = friction rub) and dyspnea score (dashed line; 0 = no dyspnea, 1 = dyspnea on exertion, 2 = dyspnea at rest, 3 = severe dyspnea) are shown for the three PBS-treated control animals (#1 to #3) and the six FLUAVsw-infected animals (#4 to #9). **(B)** Weekly collected serum samples were tested for H1N2-neutralizing antibodies by serum neutralization tests. Titers are expressed as the reciprocal neutralizing dose 50 (ND50). Data of PBS-treated (#1 to #3) and FLUAVsw-infected (#4 to #9) animals is shown in the time course following infections. (A and B) The background color in each diagram indicates different time spans: pre-infection (white), post primary infection (light grey) and post secondary infection (dark grey).

Typically, influenza infection of pigs leads to a rapid onset of respiratory signs accompanied by fever and lethargy. To verify successful influenza infection and to evaluate protection from reinfection, animals were monitored daily for clinical signs of disease, including inspection, lung auscultation and measurement of rectal temperature. A clinical score was used to specify quality and severity of the observed signs. PBS-treated control animals (animal #1 to #3) did not show any signs of illness throughout the entire study (Figure 1A, first column and data not shown). All infected animals showed lethargy, fever (>40 °C, Additional file 2) and respiratory signs, including nasal discharge, coughing, dyspnea and abnormal lung auscultation, starting one day post intratracheal administration of virus. Exemplary scores for dyspnea and lung auscultation of all infected animals (#4 to #9) are displayed in Figure 1A. Severity and quality of these lower respiratory tract signs were similar among individuals, with the exception of animal #7, showing severe dyspnea and pleural friction rub in lung auscultation for several days following infection. All animals recovered within 7 to 9 days and did neither show clinical signs nor fever after the second infection. At necropsy, six (#4, #6, #8) and nine (#5, #7, #9) weeks post primary infection, the lungs of animals #4, #7, #8 and #9 showed localized lobular atelectasis with fibrous retraction at the edges of the cranial and middle lobes. Enlarged bronchial or mediastinal lymph nodes were found in animals #5, #6, #7, #8 and #9. Animal #7 additionally showed severe adhesive pleuritis and pericarditis. Animals #5 and #6 were without macroscopic changes. Compared to control animals, histopathology revealed increased peribronchial and interstitial infiltration of mononuclear cells in animals #4, #5, #8 and #9. Retracted lobules showed atelectasis with interstitial fibrosis, metaplasia of alveolar epithelium as well as hyperplastic bronchiolar epithelium (Additional file 3). In animal #7, in addition to increased mononuclear interstitial infiltration and BALT hyperplasia, histopathology showed multifocal purulent bronchopneumonia.

### H1N2-neutralizing antibodies

Neutralizing antibodies are considered as a hallmark for strain-specific immunity following influenza infection. We analyzed H1N2-neutralizing antibodies in sera of infected and non-infected animals on a weekly basis. As displayed in Figure 1B, neutralizing antibodies were present in all infected animals (#4 to #9) one week after primary infection. Slightly higher titers were measured two weeks after primary infection. A boost of neutralizing antibodies following the second infection could be observed in animals #5, #6, #8 and #9. In all animals, neutralizing antibodies were detectable until euthanasia, 6 and 9 weeks after primary infection. Control animals (#1 to #3) were negative for neutralizing antibodies against H1N2 and all animals were negative for neutralizing antibodies against selected H1N1, H3N2 and panH1N1 strains throughout the study (data not shown).

### Ex vivo Ki-67 expression

**Figure 2:**
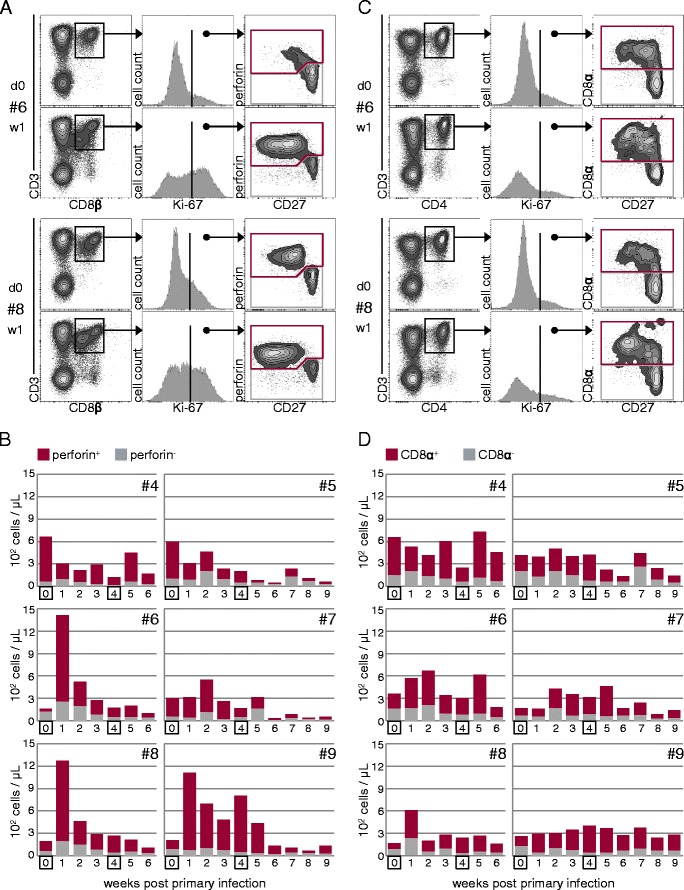
**Kinetics of Ki-67** ^**+**^ **CD8β** ^**+**^ **and Ki-67** ^**+**^ **CD4** ^**+**^ **T** **cells in peripheral blood of FLUAVsw-infected pigs.** Freshly isolated PBMCs were stained and analyzed for CD4, CD8α, CD8β, CD27, Ki-67 and perforin expression by FCM. **(A)** CD3^+^CD8β^+^ T cells were gated for Ki-67 expression and Ki-67^+^ cells were further analyzed for expression of CD27 and perforin. Perforin^+^ (red gate) and perforin^−^ (grey gate) Ki-67^+^CD3^+^CD8β^+^ T cells were further subgated for quantitative analysis over the time course following infection. **(C)** CD3^+^CD4^+^ T cells were gated for Ki-67 expression and Ki-67^+^ cells were further analyzed for CD8α and CD27 expression. CD8α^+^ (red gate) and CD8α^−^ (grey gate) Ki-67^+^CD3^+^CD4^+^ T cells were subgated for quantitative analysis over the time course following infection. **(A and C)** Exemplary raw data of animals #6 and #8, prior to infection and one week post infection, is shown. **(B and D)** Absolute numbers of Ki-67-expressing subsets within CD8β^+^ T cells (B) and CD4^+^ T cells (D) of six infected animals in the time course following infection. For calculation of absolute numbers, total lymphocyte counts were multiplied by percent values obtained by gating on the respective T-cell subpopulation.

Ki-67 expression analysis has been used in human immunological studies to detect and quantify the expansion phase of T cells responding to vaccination against smallpox [17], yellow fever [17,18], but also infection with hantavirus [19] and H1N1 influenza A virus [20]. Similarly, we hypothesized that infection of pigs with FLUAVsw may lead to increased frequencies of blood-derived Ki-67^+^ T-cell subsets in the time course following infection. Weekly, freshly isolated PBMCs were stained for Ki-67 expression in combination with different T-cell lineage and differentiation markers and analyzed by FCM. Peripheral blood TCR-γδ^+^ T cells, analyzed for CD2, CD8α, and CD27 expression, did not show any phenotypic changes or any increase in the absolute number of Ki-67 expressing cells following infection (data not shown). CD8β^+^ T cells, which represent porcine cytolytic T cells [21], were analyzed for the expression of perforin and CD27. By gating on CD27^dim/-^perforin^+^ and CD27^high^perforin^−^ Ki-67^+^CD8β^+^ T cells, we distinguished perforin^−^ and perforin^+^ CD8β^+^ T cells (Figure 2A) and calculated absolute numbers in the time course following infection (Figure 2B). In three animals (#6, #8 and #9) we could detect a marked increase of Ki-67^+^CD8β^+^ T cells one week after primary infection followed by a continuous decline in weeks 2 and 3. The expansion and contraction was mainly attributable to perforin^+^Ki-67^+^CD8β^+^ T cells. This might indicate an infection-related expansion of CD8β^+^ T cells in at least three (#6, #8 and #9) out of six animals. However, following the second infection, no increase of Ki-67^+^CD8β^+^ T cells could be detected in these three animals, and only a slight increase was found in animals #4 and #7.

In parallel, we analyzed CD4^+^ T cells for FLUAVsw infection-related changes in Ki-67 expression. Expression of CD8α and CD27 were co-analyzed on Ki-67^+^CD4^+^ T cells (Figure 2C). CD8α is expressed on activated and memory CD4^+^ T cells in swine [22,23] and down-regulation of CD27 has recently been described as a means to identify effector memory cells within porcine CD4^+^CD8α^+^ T cells [24]. By respective gates, Ki-67^+^CD4^+^ T cells were separated into naïve CD8α^−^ cells and antigen-experienced CD8α^+^ cells and, again, absolute numbers were calculated in the time course following infection (Figures 2C and D). In animals #6, #7 and #8, we observed a slight increase of Ki-67^+^CD4^+^ T cells, one or two weeks after primary infection, with the majority of proliferating cells expressing the activation marker CD8α. Following the second infection, an increase of Ki-67^+^CD4^+^ T cells could be detected in animals #4, #6 and #7. However, overall, no substantial increase, as seen for CD8β^+^ T cells in animals #6, #8 and #9, was found for the CD4^+^ T-cell population.

### IFN-γ ELISpot

**Figure 3:**
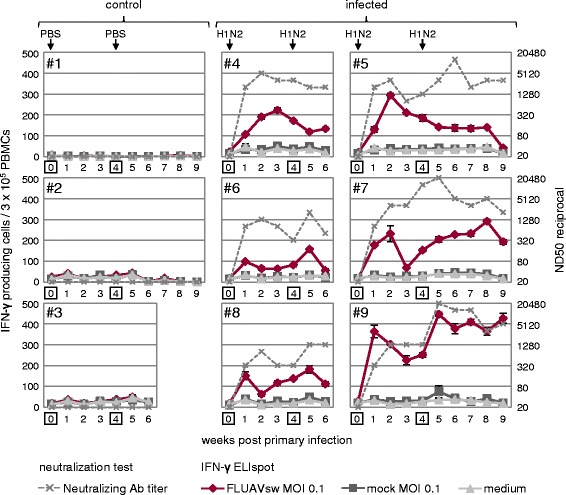
**Kinetics of FLUAVsw-specific IFN-γ-producing T cells identified by ELISpot and H1N2-neutralizing antibody titers.** For IFN-γ ELISpot, freshly isolated PBMCs were in vitro restimulated for 24 h with FLUAVsw (infection strain; MOI = 0.1). Mock- and medium-incubated cultures served as negative controls. Frequency of IFN-γ-producing cells within 3 × 10^5^ PBMCs is displayed for FLUAVsw- (red line), mock- (dark grey line) and medium-incubated (light grey line) cultures. Neutralizing antibody titers are displayed in dashed grey lines according to the secondary y-axis (for details see caption of Figure 1B). Data of PBS-treated (#1 to #3) and FLUAVsw infected (#4 to #9) animals is shown in the time course following infections.

Ex vivo Ki-67 expression kinetics of PBMCs pointed towards increased proliferative activity of T cells following FLUAVsw infection, but the specificity of this response remains uncertain. As a first approach to identify FLUAVsw-specific lymphocyte responses within PBMCs, we measured IFN-γ production in ELISpot assays following in vitro restimulation with homologous virus. As displayed in Figure 3, background levels of around 40 IFN-γ producing cells per 3 × 10^5^ PBMCs were found in microcultures stimulated with mock supernatants or cell culture medium at all investigated time points (dark grey and light grey lines). Similar levels were found in virus-stimulated microcultures with PBMCs from non-infected control animals. Within PBMCs of all six infected animals, FLUAVsw-specific IFN-γ-producing cells could be detected already one week after primary infection (Figure 3, red lines). The magnitude of the IFN-γ response differed between individuals, with animal #9 having up to four times as many IFN-γ producing cells per 3 × 10^5^ PBMCs as compared with the other animals. This difference was especially pronounced in week 1 after primary infection and in week 5, when animal #9 showed a clear booster response following the second infection. Booster responses were also found in PBMCs of animals #6 and #8. For an immediate comparison of the IFN-γ response in ELISpot assays and the neutralizing antibody titers, the trajectory curves of Figure 1B have been plotted into Figure 3. Overall, some synchronicity between the frequency of IFN-γ producing cells and antibody titers could be observed (animals #4, #6, #8 and #9), although there were opposing courses at certain periods or time points.

### IFN-γ, TNF-α and IL-2 production by CD4^+^ T cells

**Figure 4:**
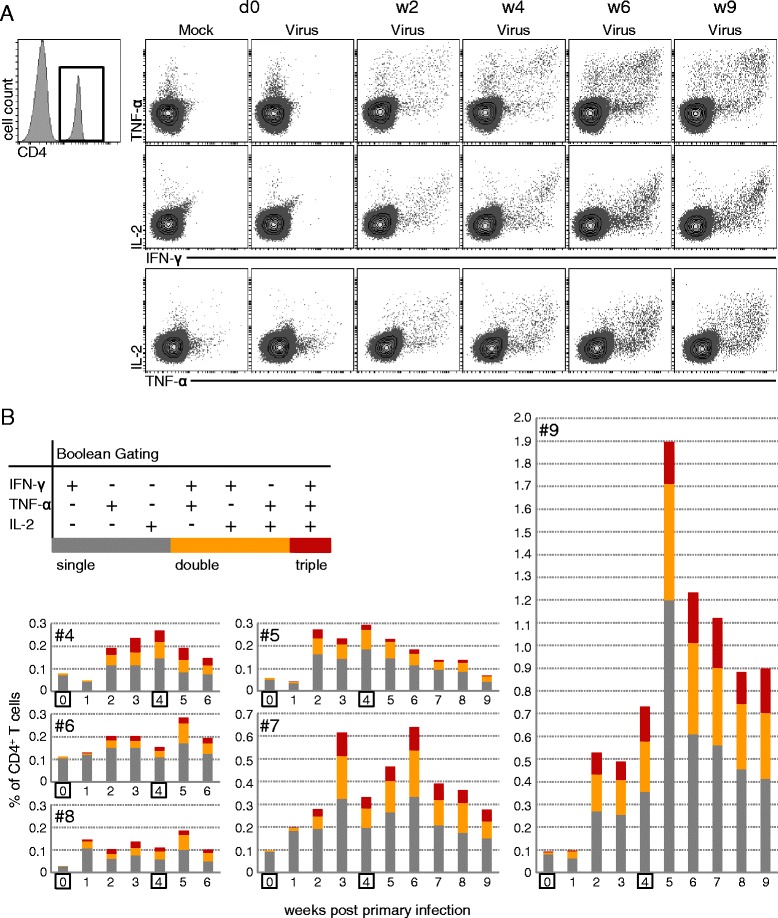
**Kinetics of FLUAVsw-specific IFN-γ-/TNF-α-/IL-2-producing CD4** ^**+**^ **T cells.** Intracellular cytokine staining of defrosted PBMCs was performed following overnight in vitro restimulation with FLUAVsw (infection strain, MOI = 0.1; 18 h). Mock-incubated cultures served as negative controls. **(A)** CD4^+^ T cells were gated and analyzed for production of IFN-γ, TNF-α and IL-2. Contour plots show combinations of cytokines for selected time points following FLUAVsw infection. Exemplary data of animal #9 is shown. **(B)** Boolean gating was applied in order to identify single-, double-, and triple-cytokine-producing CD4^+^ T cells. Single- (dark grey), double- (orange) and triple- (red) cytokine-producing cells are shown as percent of total CD4^+^ T cells for all six infected animals in the time course following FLUAVsw infection.

As IFN-γ ELISpots indicated the presence of FLUAVsw-specific lymphocytes within PBMCs of infected animals, the next step was to assign a phenotype to these responding cells. On top, we aimed to identify potential multifunctional FLUAVsw-specific T cells, as the simultaneous production of different cytokines or effector molecules on the single T-cell level is proposed to be a hallmark of protective immune responses [25]. Accordingly, we performed intracellular cytokine staining for IFN-γ, TNF-α and IL-2 in CD4^+^ and CD8β^+^ T cells following FLUAVsw in vitro restimulation of PBMCs. As displayed in Figure 4A and Additional file 4, within CD4^+^ T cells, double-cytokine-producing cells were virtually absent prior to infection, but could be identified from week 2 after primary infection onwards. Boolean gating was used to quantify single-, double- and triple-cytokine-producing CD4^+^ T cells in the time course following infection, as displayed in Figure 4B. A detailed analysis of the frequency of CD4^+^ T cells producing a particular single cytokine or cytokine combination is shown in Additional file 5. Overall, FLUAVsw-specific cytokine^+^CD4^+^ T cells were found to be increased from one (#7, #8) or two (all other animals) weeks after primary infection. Double- and triple-cytokine-producing cells started to be detectable at two weeks after primary infection. In animal #9, we observed a massive increase in the frequency of cytokine^+^CD4^+^ T cells at one week post secondary infection (i.e. five weeks after primary infection). This increase was attributable mainly to IFN-γ single-producing and IFN-γ/TNF-α double-producing CD4^+^ T cells (Additional file 5). Apart from animal #9, an increase of cytokine^+^CD4^+^ T cells following the second infection could be detected in animals #6, #7 and #8. Single-cytokine-producing CD4^+^ T cells produced mainly IFN-γ or TNF-α. Double-cytokine-producing CD4^+^ T cells predominantly produced IFN-γ in combination with TNF-α (Additional file 5).

**Figure 5:**
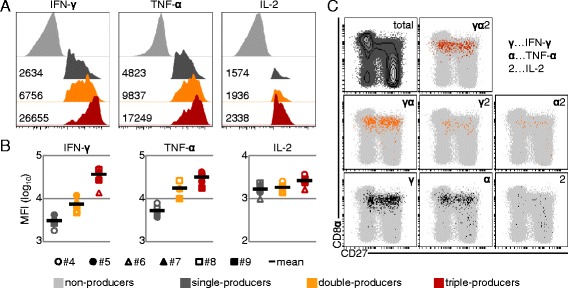
**IFN-γ/TNF-α/IL-2 expression levels and CD8α/CD27 expression of cytokine-producing CD4** ^**+**^ **T cells.** (A + B) Fluorescence intensity of IFN-γ, TNF-α and IL-2 expression within single- (dark grey), double- (orange) and triple- (red) cytokine-producing CD4^+^ T cells. **(A)** Histograms show exemplary data of animal #9 at six weeks post primary infection. Numbers indicate median fluorescence intensity (MFI) for each subset and the respective cytokine. **(B)** MFI of IFN-γ, TNF-α and IL-2 expression within single- (dark grey), double- (orange) and triple- (red) cytokine-producing CD4^+^ T cells isolated at six weeks post primary infection. Data of all six infected animals is shown by individual symbols. The mean is indicated by the black bar. **(C)** CD8α and CD27 expression on total CD4^+^ T cells (contour plot in the upper left and light grey dots in dot plots) and CD4^+^ T cells producing a single cytokine (dark grey dots, bottom), two cytokines (orange dots, middle) or three cytokines (red dots, top). Representative raw data of animal #9, six weeks post primary infection, is shown.

Studies in humans and mice have shown that multiple-cytokine-producing T cells produce more cytokines on a per-cell basis compared to single-cytokine producers [26-28]. By comparing the median fluorescence intensity (MFI) obtained by FCM for the investigated cytokines (IFN-γ, TNF-α, IL-2) among single-, double- and triple-cytokine-producing CD4^+^ T cells isolated from week 2 after primary infection onwards, we found higher quantities of IFN-γ and TNF-α in triple- and double-producers (Figures 5A and B and Additional file 6). IL-2 was slightly increased in triple-producers, compared with single- and double-producers. Overall, these findings might support the hypothesis that multiple-cytokine-producing FLUAVsw-specific T cells have a beneficial role in clearance of the infection.

Previous work of our group suggested that porcine CD4^+^CD8α^+^ T cells can be differentiated upon their expression of CD27, with CD27^+^ and CD27^−^ cells resembling central memory (T_CM_) and effector memory T helper cells (T_EM_), respectively [24]. Therefore, we investigated the seven different cytokine-producing subsets identified by boolean gating for expression of CD8α and CD27. As shown in Figure 5C and Additional file 7, the vast majority of cytokine^+^CD4^+^ T cells expressed CD8α (with some notable exceptions of TNF-α and IL-2 single-producing cells), thereby displaying the phenotype of activated or antigen-experienced porcine CD4^+^ T cells. However, within these CD4^+^CD8α^+^ T cells, the CD27 expression profile did not allow for a clear assignment of responding cells to the CD27^−^ T_EM_ or the CD27^+^ T_CM_ subset, as all seven cytokine-producing subsets were distributed among both populations (Figure 5C and Additional file 7). In summary, these data indicate that CD4^+^ T cells are involved in the immune response to influenza infection in pigs and that a considerable proportion of responding cells is multifunctional in terms of IFN-γ, TNF-α and IL-2 production.

### Proliferation, degranulation and cytokine production by CD8β^+^ T cells

**Figure 6:**
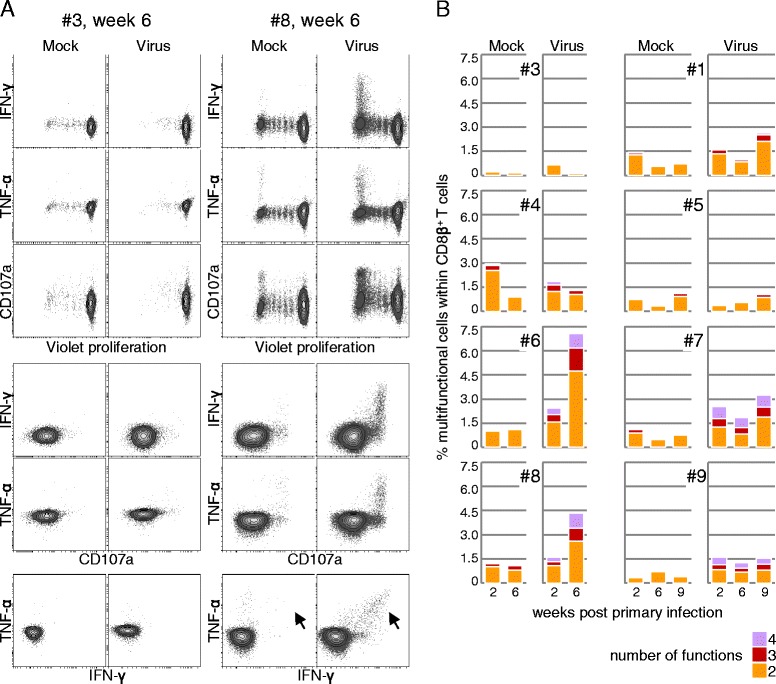
**Proliferation, degranulation and cytokine production by FLUAVsw-specific CD8β** ^**+**^ **T cells.** Violet-labeled PBMCs isolated at 2, 6 and 9 weeks post primary infection were restimulated twice with FLUAVsw (infection strain, MOI = 0.1; day 0 and day 5 of culture) and analyzed by FCM on day 6 of culture. **(A)** CD8β^+^ T cells were gated (not shown) and analyzed for violet proliferation, surface CD107a expression and intracellular accumulation of IFN-γ and TNF-α. Contour plots show all possible marker combinations. Exemplary raw data of one non-infected control animal (#3) and one infected animal (#8) is shown for week 6 post primary infection. **(B)** Boolean gating was applied in order to identify multifunctional CD8β^+^ T cells, and resulting CD8β^+^ T-cell subsets were grouped by number of functions. Displayed is the frequency of CD8β^+^ T cells carrying out two or more functions within total CD8β^+^ T cells. Data of six FLUAVsw-infected animals (#4-9) and two non-infected control animals (#1, #3) is shown.

Having observed typical clinical signs of FLUAVsw infection, indicative of active viral replication in the upper and lower respiratory tract, we expected to see a FLUAVsw-specific activation of MHC-I-restricted CD8β^+^ T cells in infected pigs. As shown above, analysis of ex vivo Ki-67 expression in peripheral blood CD8β^+^ T cells revealed expression dynamics reminiscent of an expansion and contraction phase of effector CD8β^+^ T cells in at least three out of six animals. Using the overnight restimulation protocol established for the intracellular cytokine staining of CD4^+^ T cells (Figure 4), we detected very low frequencies of FLUAVsw-specific cytokine-producing CD8β^+^ T cells (Additional file 8). With the aim to increase the frequency of FLUAVsw-specific CD8β^+^ T cells by in vitro expansion, we developed an alternative stimulation protocol, consisting of a 6-day culture of PBMCs, with a first FLUAVsw restimulation on day 0 and a second FLUAVsw restimulation on day 5 of culture. This prolonged incubation time enabled us to additionally investigate proliferation in mock- and FLUAVsw-stimulated cultures by the use of violet proliferation dye. Moreover, as hardly any IL-2^+^CD8β^+^ T cells could be detected by the overnight restimulation (Additional file 8), we decided to omit IL-2 and to analyze surface CD107a expression, indicative of degranulation, instead. PBMCs isolated at two weeks after primary infection, six weeks after primary infection (i.e. two weeks after secondary infection) and – where available – nine weeks after primary infection (i.e. five weeks after secondary infection) were analyzed. For each time point, intracellular IFN-γ and TNF-α, surface CD107a expression and violet proliferation were analyzed. Exemplary contour plots displaying all possible IFN-γ/TNF-α/CD107a/proliferation-marker combinations of gated CD8β^+^ T cells are shown for one control animal (#3) and one infected animal (#8) at six weeks after primary infection (Figure 6A). CD8β^+^ T cells of non-infected control animals showed some proliferation and degranulation, but hardly any cytokine production in response to FLUAVsw or mock stimulation. Within CD8β^+^ T cells of infected animals, cytokine production was detected only in cells that had proliferated and all cytokine^+^ cells also stained positive for the degranulation marker CD107a (Figure 6A). Importantly, IFN-γ/TNF-α double positive CD8β^+^ T cells were absent in mock-stimulated cultures, but clearly detectable upon FLUAVsw restimulation (Figure 6A, arrows). Having measured four different functions of CD8β^+^ T cells simultaneously, we applied boolean gating to identify and quantify all subsets, differing in the type and number of functions. From 11 possible multifunctional subsets (i.e. ≥2 functions), three subsets dominated strongly: proliferating CD107^+^ cells, (two functions), proliferating CD107^+^IFN-γ^+^ cells (three functions) and proliferating CD107^+^IFN-γ^+^TNF-α^+^ cells (four functions; Additional file 9). Therefore, the subsets were clustered according to the number of functions they exerted (Figure 6B). Results are displayed as percent of multifunctional cells within total CD8β^+^ T cells. Animals #6 and #8 showed the highest frequency of FLUAVsw-specific multifunctional cells within CD8β^+^ T cells, isolated at six weeks after primary infection, i.e. two weeks after secondary infection. A lower frequency was detected in animals #7 and #9 and no clear increase could be detected from the earliest to later time points. Hardly any virus-specific response could be detected in CD8β^+^ T cells of animals #4 and #5, similar to the non-infected control animals (#1, #3). Notably, the lacking FLUAVsw-specific CD8β^+^-T-cell response of animals #4 and #5 matched the results of the Ki-67 expression analysis (Figure 2B), as no expansion of Ki-67-expressing CD8β^+^ T cells could be detected in these two animals. In summary, these data indicate the presence of FLUAVsw-specific multifunctional CD8β^+^ T cells within PBMCs of four out of six animals.

### SLA haplotyping

T-cell responses are influenced by the binding properties of particular major histocompatibility complex molecules that mediate the presentation of peptides to the T-cell receptor. To identify potential associations between the T-cell response and the SLA haplotype, a low-resolution SLA typing of the nine pigs included in the present study was performed and revealed a total of nine class I and eight class II haplotypes (Table 2). The most common class I haplotypes were Lr-01.0 (#4, #6, #7, #9) and Lr-24.0 (#3, #5, #6, #7), followed by Lr-25.0 (#1, #4) and Lr-59.0 (#2, #3). Unique class I haplotypes were Lr-45.0 (#1), Lr-08.0 (#2), Lr-18.0 (#5), Lr-40.0 (#8) and Lr-46.0 (#8). Within class II, the most frequent haplotype was Lr-0.15b (#2, #3, #4, #5, #6, #7), followed by Lr-0.09 (#3, #5, #6, #7) and Lr-0.25 (#1, #2, #4). Unique class II haplotypes were found in pig #1 (Lr-0.22), pig #8 (Lr-0.10/Lr-0.23) and pig #9 (Lr-0.33/Lr-0.35).

**Table 2:** **Swine leukocyte antigen (SLA) haplotypes of pigs used in this study**

		**SLA allele specificity**				**SLA allele specificity**		
**Pig #**	**SLA class I haplotype**	**SLA-1**	**SLA-3**	**SLA-2**	**SLA class II haplotype**	**SLA-DRB1**	**SLA-DQB1**	**SLA-DQA**
1	Lr-25.0	11XX	03XX	07XX	Lr-0.22	06XX	02XX(0204)	02XX
	Lr-45.0	08XX + cs02	07XX	w08XX + 10XX	Lr-0.25	13XX	09XX	04XX
2	Lr-08.0	04XX	03XX	07XX	Lr-0.15b	04XX	02XX	02XX
	Lr-59.0	11XX(1103)	05XX	jh02	Lr-0.25	13XX	09XX	04XX
3	Lr-24.0	blank	04XX/hb06	02XX	Lr-0.09	02XX	04XX	03XX
	Lr-59.0	11XX(1103)	05XX	jh02	Lr-0.15b	04XX	02XX	02XX
4	Lr-01.0	01XX	01XX	01XX	Lr-0.15b	04XX	02XX	02XX
	Lr-25.0	11XX	03XX	07XX	Lr-0.25	13XX	09XX	04XX
5	Lr-18.0	04XX	03XX	01XX	Lr-0.09	02XX	04XX	03XX
	Lr-24.0	blank	04XX/hb06	02XX	Lr-0.15b	04XX	02XX	02XX
6	Lr-01.0	01XX	01XX	01XX	Lr-0.09	02XX	04XX	03XX
	Lr-24.0	blank	04XX/hb06	02XX	Lr-0.15b	04XX	02XX	02XX
7	Lr-01.0	01XX	01XX	01XX	Lr-0.09	02XX	04XX	03XX
	Lr-24.0	blank	04XX/hb06	02XX	Lr-0.15b	04XX	02XX	02XX
8	Lr-40.0	16XX	05XX	10XX	Lr-0.10	04XX	08XX	03XX
	Lr-46.0	12XX	04XX/hb06	06XX	Lr-0.23	10XX/er01	06XX/zs12	01XX
9	Lr-01.0	01XX	01XX	01XX	Lr-0.33	11XX	02XX	02XX
	Lr-01.0	01XX	01XX	01XX	Lr-0.35	01XX	04XX	02XX

## Discussion

In the present study, we aimed to investigate the kinetics and functional properties of FLUAVsw-specific CD4^+^ and CD8β^+^ T cells in the blood of FLUAVsw-infected pigs. We also decided to perform a homologous reinfection four weeks after the primary infection in order to monitor a potential expansion or change in functional attributes of FLUAVsw-specific memory T cells under such conditions. A FLUAVsw H1N2 isolate from Germany was used for infection and in vitro restimulation. H1N2 virus strains are constantly circulating in European pig herds since the 1990s [4]; therefore our infection model should at least partially reflect field conditions.

To verify successful experimental infection with the intratracheally administered H1N2 isolate, clinical signs and post-mortem histopathological changes were examined. All infected animals developed clinical signs with fever, lethargy and lower-respiratory-tract signs, which have been described as the typical outcome of acute influenza infection [4,29]. Within one week, all animals recovered, which is consistent with the reported clearance of influenza virus from the porcine lung within seven days [4]. Clinical signs were absent from all animals upon reinfection with the same strain four weeks later. This is in accordance with previous studies, where pigs underwent a homologous reinfection [5,7,30,31]. Macroscopic lesions were either mild, and confined to localized lobular retractions at the edges of the cranial lobes, or were even absent. Microscopically, we observed mild to moderate infiltration of the lung tissue with mononuclear cells and lobular atelectasis with fibrous retraction as well as metaplastic and hyperplastic changes of the airway epithelium. These lesions were considered as signs of late-stage influenza infection. The microscopic lesions observed in animal #7 resembled alterations present in intercurrent bacterial infections (such as *Mycoplasma* spp., *Pasteurella multocida* or *Haemophilus parasuis*) and were not representative for the infection group.

H1N2-neutralizing antibodies were detected at considerable levels already one week after infection, confirming the postulated relevance of the humoral response in the clearance of FLUAVsw infections. With the exception of animal #9, a clear boost in antibody titers was not observed, which is consistent with a study reporting the lack of a HI-titer increase after homologous reinfection [5].

As a first attempt to analyze the role of T cells in the control of FLUAVsw infection, we looked for expansion of CD4 and CD8β effector T cells via analysis of Ki-67 expression. In parallel, we followed the frequency of FLUAVsw-specific multiple-cytokine-producing T cells. The analysis of Ki-67^+^ T cells has been successfully used in immunological studies addressing T-cell expansion in response to influenza A virus infections within PBMCs isolated from humans [20] and nonhuman primates [32]. In our study, we could identify a clear expansion of CD8β^+^Ki-67^+^perforin^+^ T cells at one week after primary infection in three out of six animals. This heterogeneity is somewhat similar to results obtained by a study of human T cells [20], where, seven days after infection, a strong variation in the frequency of CD4^+^CD38^+^Ki-67^+^ and CD8^+^CD38^+^Ki-67^+^ T cells was observed between different individuals. In contrast, a more homogenous response in regard to expansion of blood-derived CD8^+^CD38^+^Ki-67^+^ T cells has been reported for H1N1-infected rhesus macaques [32]. The reasons for the lack of a CD8β^+^-T-cell expansion in the blood of at least two infected pigs (#4, #5) are speculative. Interestingly, these two animals also lacked a response of CD8β^+^ T cells in the in vitro restimulation experiments addressing FLUAVsw-specific proliferation, cytokine production and CD107a expression (Figure 6B). These findings may suggest that a CTL response was only weakly triggered in these two animals. Clearly, a parallel analysis of the T-cell response present in the lung will lead to a more comprehensive overview of the cell-mediated response and its variation among individual pigs.

The lack of a substantial FLUAVsw infection-related expansion of CD4^+^ T cells (in all animals) might be also due to a masking by T-cell responses to other environmental antigens encountered following weaning, and by T-cell responses to the vaccines (porcine circovirus type 2, *Mycoplasma hyopneumoniae*, see chapter 2.1) administered at three weeks of age. Indeed, in a previous study from our group we could show that, following weaning at three weeks of age, a considerable increase of CD4^+^ T cells with an activated and/or memory phenotype occurs in blood [23].

These observations also provide some evidence that, despite the relatively young age of the piglets in this study at the time of the first infection (i.e. four weeks), CD4^+^ T cells were already capable to respond to the FLUAVsw infection. Similarly, the observed expansion of CD8β^+^ T cells in the blood of three animals following FLUAVsw infection might indicate that also this T-cell subset was already in a maturation stage enabling a reaction to this viral infection.

Multifunctional T cells have been proposed as a hallmark of protective immunity [18,26,27,33,34]. Performing triple-cytokine staining of in vitro restimulated PBMCs, we found that a considerable proportion of FLUAVsw-specific CD4^+^ T cells can be activated to produce two or three of the cytokines IFN-γ, TNF-α and IL-2 simultaneously. Triple-cytokine-producing CD4^+^ T cells were detectable in the blood of all infected pigs from two weeks post primary infection onwards. This rather late appearance in regard to the clearance of clinical signs questions their immediate role in the primary immune response against FLUAVsw, but may indicate relevance in the formation of memory T cells. Also, an increase of single-, double- and triple-cytokine-producing CD4^+^ T cells was observed in four (#6, #7, #8, #9) out of six animals after the secondary infection, supporting the assumption that multifunctional CD4^+^ T cells are a part of the FLUAVsw-specific memory-T-cell pool.

CD4^+^ multifunctional T cells produced more IFN-γ, TNF-α and IL-2 on a per-cell level, and triple producers outranked double-producers, especially in the production of IFN-γ (Figures 5A and B and Additional file 6). This confirms an observation already made for murine [26,28] and human T cells [27,34,35]. For CD8β^+^ T cells, IL-2 production was undetectable in the overnight restimulated cultures (Additional file 8). Interestingly, recent work from our laboratory showed that, even after polyclonal stimulation, CD8β^+^ T cells isolated from porcine blood produce hardly any IL-2, whereas such T cells can be readily identified in bronchial lymph nodes [36]. This might indicate a rapid homing of IL-2-producing CD8β^+^ T cells into lymphatic organs. Of note, human influenza-specific CD8^+^ T cells were shown to produce IL-2 upon influenza A virus restimulation, but protection from severe illness was found to correlate only with preexisting IFN-γ^+^CD8^+^ T cells and no correlation was found for IL-2-producing CD8^+^ T-cell subsets [37].

Generally, cytokine production of CD4^+^ T cells was mostly associated with CD8α expression, which is described as a marker to distinguish naïve from antigen-experienced CD4^+^ T cells in swine [22,23]. Cytokine combinations including IFN-γ appeared to be even more restricted to CD8α expression, which is in accordance with the description of IFN-γ being a signature cytokine of late-differentiated human T cells [38]. For CD27 expression, which was proposed to distinguish between effector and central memory CD4^+^ T cells in swine [24], we could not find any cytokine-related patterns. Both, the putative CD8α^+^CD27^+^ central memory subset and the putative CD8α^+^CD27^−^ effector memory subset contained FLUAVsw-specific cytokine-producing T cells, indicating that both T-cell subsets contribute to the FLUAVsw-specific memory-T-cell pool in pigs.

A reliable frequency of FLUAVsw-specific CD8β^+^ T cells could only be identified after a six-day in vitro expansion. These responding CD8β^+^ T cells consisted mainly of IFN-γ/TNF-α-double-producing cells (Figure 6A, arrows). A dominant co-production of IFN-γ and TNF-α was also shown for human CD8^+^ T cells responding to influenza peptide restimulation [39]. This co-production of the antiviral cytokines IFN-γ and TNF-α is described as the predominant functional signature of effector CD8^+^ T cells in humans [38].

Similar to the variable frequencies of FLUAVsw-specific cytokine-producing CD4^+^ T cells, we found considerable variation in magnitude and kinetics of FLUAVsw-specific cytokine-producing CD8β^+^ T cells among individual pigs (Figure 6B). Strong variations of the CD8^+^-T-cell response were also described for outbred mice following bacterial or viral infection [40]. In humans, a recent study of CD8^+^-T-cell immunity across ethnicities highlighted the influence of different human leukocyte antigen class 1 haplotypes on the resulting individual differences in magnitude and quality of influenza-specific CD8^+^ memory T cells [39]. Interestingly, the low-resolution swine leukocyte antigen (SLA) typing of the pigs used in our study revealed that pig #9, which showed a strong expansion of Ki-67^+^CD8β^+^ T cells and the highest frequencies of FLUAVsw-specific cytokine-producing CD4^+^ T cells, had a homozygous SLA class I haplotype and was also homozygous for the SLA-DQA locus (Table 2). However, the SLA class I haplotype of animal #9 was also present in the CD8β^+^-T-cell-non-responding pig #4, indicating that further factors or minor differences in the SLA class I haplotypes, only identifiable by high-resolution SLA typing, are responsible for the observed differences in the CD8β^+^-T-cell response.

In conclusion, our data demonstrate the generation of FLUAVsw-specific CD4^+^ and CD8β^+^ T cells in the immune response to influenza A virus infection in the pig. The observed multifunctional profile of FLUAVsw-specific T cells suggests a function in protective immunity, similar to the relevance of multifunctional T cells in the control of viral infections in humans [25]. Clearly, this has to be addressed in heterologous reinfection studies, which will provide important insights into T-cell- mediated cross-protection. Of note, the T-cell responses described in this study were analyzed in blood, i.e. on a systemic level. In future studies it is necessary to analyze FLUAVsw-specific T cells also on the local level in the lung, as FLUAVsw infections do not cause viremia [4] and therefore represent a local infection. Work on this is in progress in our laboratory, indicating a strong enrichment of FLUAVsw-specific CD8β^+^ T cells in the porcine lung (S.C. Talker and W. Gerner, unpublished data). Such an in-depth characterization of the porcine T-cell response to influenza infection will contribute to the development of improved vaccines for pig husbandry. These novel vaccines should also reduce viral transmission between swine and humans, a risk factor associated with the emergence of – potentially pandemic – reassortant influenza strains.

## Additional files

Additional file 1:
**FCM gating strategy used for freshly isolated PBMCs (A) and for defrosted, in vitro restimulated PBMCs (B).** (A + B) Lymphocytes were gated according to FSC-A/SSC-A characteristics. Consecutive FSC-H/FSC-W and SSC-H/SSC-W plots were used to gate on singlets. (B) For analyses with defrosted and in vitro restimulated PBMCs, dead cells were excluded by the use of the LIVE/DEAD stain Near-IR. Additional file 2:
**Rectal temperatures in the time course.** Rectal temperatures were recorded daily from one week prior to infection until the end of the study, i.e. 6 weeks or 9 weeks post primary infection. Mean values and standard deviations for the PBS-treated control pigs (grey line) and the H1N2-infected pigs (red line) are shown in the time course. Asterisks at one and two days post primary infection indicate significant differences between the infected and the control group (*p* ≤ 0.001, unpaired *t*-test, SPSS Inc./ IBM, Chicago, IL, USA, Version 19). Additional file 3:
**Representative macroscopic and histologic lesions in a FLUAVsw-infected pig (#4) in comparison to a control animal (#2).** The control animal shows no macroscopic alterations at the edges of the right cranial and middle lung lobes (A). Histologically, the lung tissue of the control animal shows thin alveolar walls and no inflammatory cells in the alveolar spaces and alveolar septa (C). The bronchioles are lined by a cuboidal epithelium and there are few mononuclear cells in the surrounding interstitium (E). In the infected animal there are several consolidated and retracted lobules at the ventral edge of the cranial and middle lobe (B). Microscopically, there is diffuse infiltration with mononuclear cells, fibrosis and metaplastic transformation of alveolar epithelia to cuboidal formations (D). Bronchiolar epithelia are markedly hyperplastic with pseudostratified columnar appearance and the surrounding interstitium is infiltrated with numerous mononuclear cells (F). Bars: 150 μm (C,D); 80 μm (E,F). Additional file 4:
**Kinetics of FLUAVsw-specific IFN-** **γ** **-/TNF-** **α** **-/IL-2-producing CD4** ^**+**^ **T cells.** Intracellular cytokine staining of defrosted PBMCs was performed following overnight in vitro restimulation with FLUAVsw (infection strain, MOI = 0.1; 18 h). Mock-incubated cultures served as negative controls. CD4^+^ T cells were gated and analyzed for production of IFN-γ, TNF-α and IL-2. Contour plots show combinations of cytokines for selected time points following FLUAVsw infection. (A) Exemplary data of animal #7 (intermediate response) and (B) animal #8 (low response) is shown. Additional file 5:
**Kinetics of FLUAVsw-specific CD4** ^**+**^ **T-cell subsets producing different cytokines.** Intracellular cytokine staining of defrosted PBMCs was performed following overnight in vitro restimulation with FLUAVsw (infection strain, MOI = 0.1; 18 h). Mock-incubated cultures served as negative controls. CD4^+^ T cells were gated and analyzed for production of IFN-γ, TNF-α and IL-2 as in Figure 4. Boolean gating was applied in order to identify all seven subsets of cytokine-producing CD4^+^ T cells. The frequency of the seven different cytokine-producing T-cell subsets are shown in stacked bar charts as percent of total CD4^+^ T cells for all six infected animals in the time course following FLUAVsw infection. Additional file 6:
**IFN-** **γ/TNF-** **α/IL-2 expression levels of cytokine-producing CD4** ^**+**^ **T cells.** (A + B) Fluorescence intensity of IFN-γ, TNF-α and IL-2 expression within single- (dark grey), double- (orange) and triple- (red) cytokine-producing CD4^+^ T cells. Histograms show data of cells isolated from animal #7 (A; intermediate response) and animal #8 (B; low response) at six weeks post primary infection. Numbers indicate median fluorescence intensity (MFI) for each subset and the respective cytokine. (C) MFI of IFN-γ, TNF-α and IL-2 expression within single- (dark grey), double- (orange) and triple- (red) cytokine-producing CD4^+^ T cells isolated at two to six weeks post primary infection. Data of all six infected animals is shown by individual symbols. The mean is indicated by the black bar. Additional file 7:
**CD8α** **/CD27 expression of cytokine-producing CD4** ^**+**^ **T cells.** CD8α and CD27 expression on total CD4^+^ T cells (contour plot in the upper left and light grey dots in dot plots) and CD4^+^ T cells producing a single cytokine (dark grey dots, bottom), two cytokines (orange dots, middle) or three cytokines (red dots, top). Data of CD4^+^ T cells from animal #7 (A; intermediate response) and animal #8 (B; low response) at six weeks post primary infection is shown. Additional file 8:
**Production of IFN-γ** **, TNF-α and IL-2 by FLUAVsw-specific CD8β** ^**+**^ **T cells.** Intracellular cytokine staining of defrosted PBMCs was performed following overnight in vitro restimulation with FLUAVsw (infection strain, MOI = 0.1; 18 h). Mock-incubated cultures served as negative controls. CD3^+^CD8β^+^ T cells were gated and analyzed for production of IFN-γ, TNF-α and IL-2. Contour plots show combinations of cytokines for selected time points following FLUAVsw infection. Exemplary data obtained with CD8β^+^ T cells of animal #6 is shown. Additional file 9:
**Multifunctional FLUAVsw-specific CD8β** ^**+**^ **T cells identified by proliferation, CD107a expression and cytokine production.** Violet-labeled PBMCs isolated at 2, 6 and 9 weeks post primary infection were restimulated twice with FLUAVsw (infection strain, MOI = 0.1; day 0 and day 5 of culture). On day 6 of culture, PBMCs were analyzed by FCM for proliferation, CD107a expression and production of IFN-γ and TNF-α. Boolean gating was applied in order to identify multifunctional subsets of CD8β^+^ T cells. All 11 multifunctional subsets (i.e. ≥ 2 functions) are shown in the left panel as stacked bar charts in % of total CD8β^+^ T cells. In the right panel, the subsets are grouped according to the number of functions they exert (as shown in Figure 6B). Data of six FLUAVsw-infected animals (#4-9) and two non-infected control animals (#1, #3) is shown.
