# Supplementary figures and images for: Magnitude and kinetics of multifunctional CD4+ and CD8β+ T cells in pigs infected with swine influenza A virus
Source: Vet Res. 2015 May 14;46(1):52. doi: 10.1186/s13567-015-0182-3 (PMC4429459; doi:10.1186/s13567-015-0182-3)

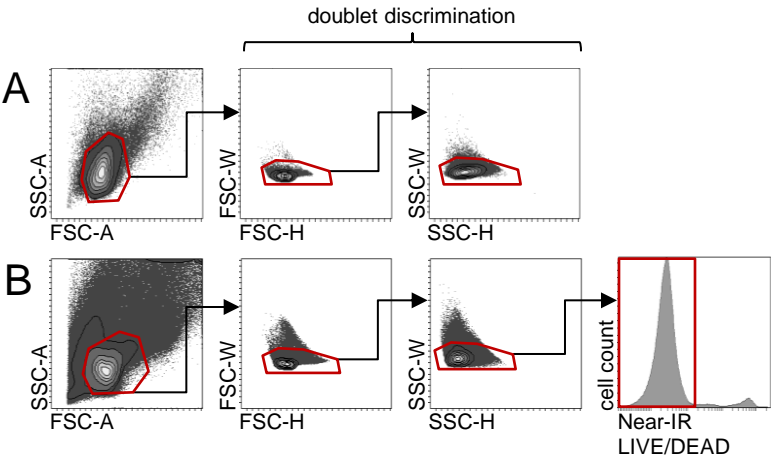

Supplement: Additional file 1: — FCM gating strategy used for freshly isolated PBMCs (A) and for defrosted, in vitro restimulated PBMCs (B). (A + B) Lymphocytes were gated according to FSC-A/SSC-A characteristics. Consecutive FSC-H/FSC-W and SSC-H/SSC-W plots were used to gate on singlets. (B) For analyses with defrosted and in vitro restimulated PBMCs, dead cells were excluded by the use of the LIVE/DEAD stain Near-IR. [file 13567_2015_182_MOESM1_ESM.pdf]

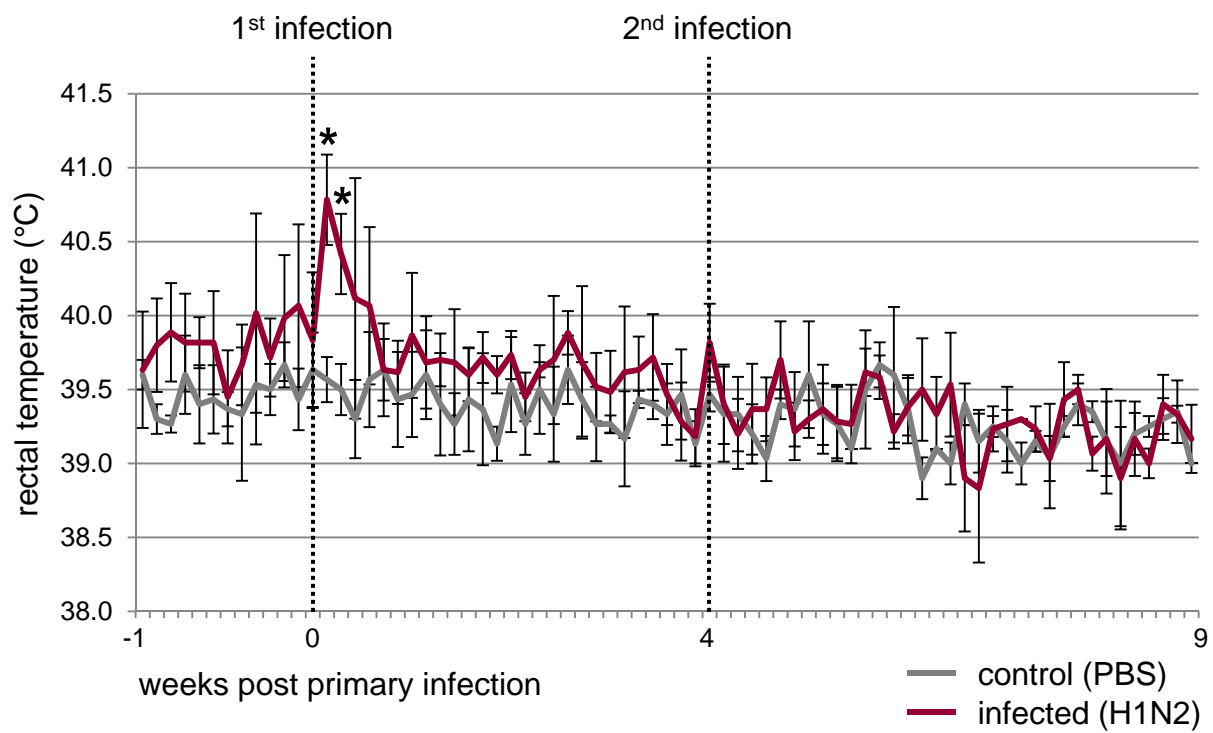

Supplement: Additional file 2: — Rectal temperatures in the time course. Rectal temperatures were recorded daily from one week prior to infection until the end of the study, i.e. 6 weeks or 9 weeks post primary infection. Mean values and standard deviations for the PBS-treated control pigs (grey line) and the H1N2-infected pigs (red line) are shown in the time course. Asterisks at one and two days post primary infection indicate significant differences between the infected and the control group (p ≤ 0.001, unpaired t-test, SPSS Inc./ IBM, Chicago, IL, USA, Version 19). [file 13567_2015_182_MOESM2_ESM.pdf]

**Control**

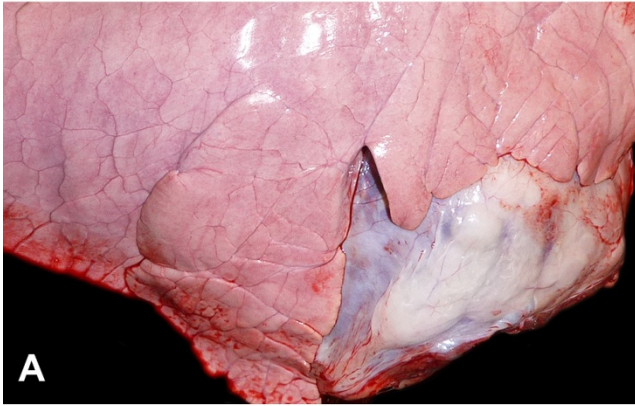

**Infected**

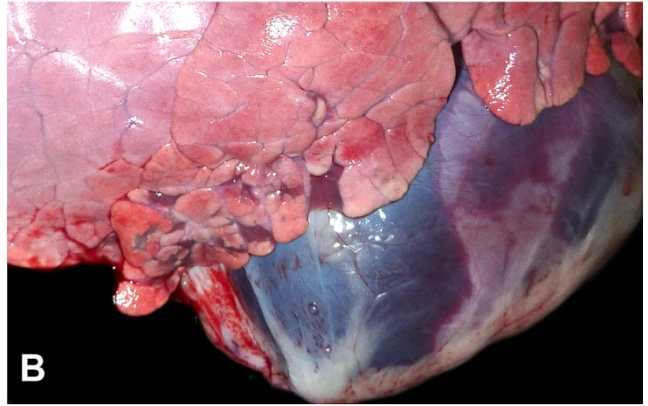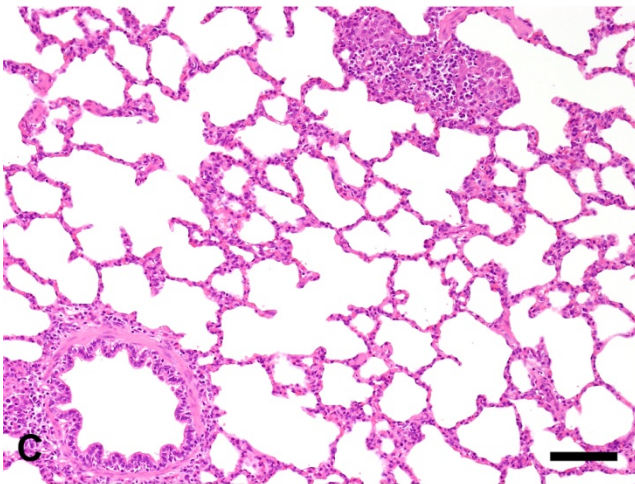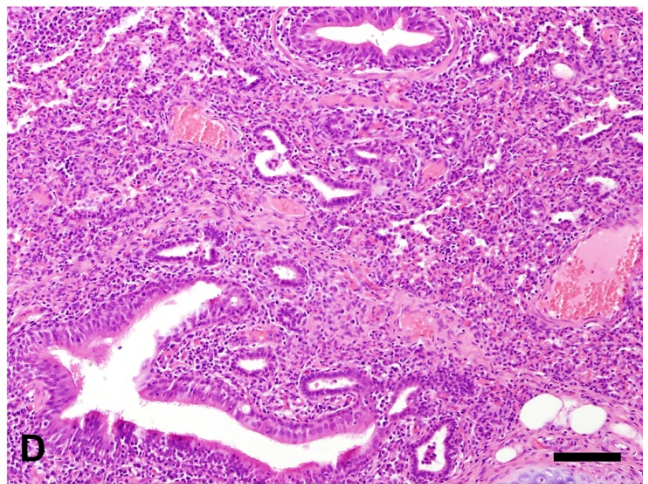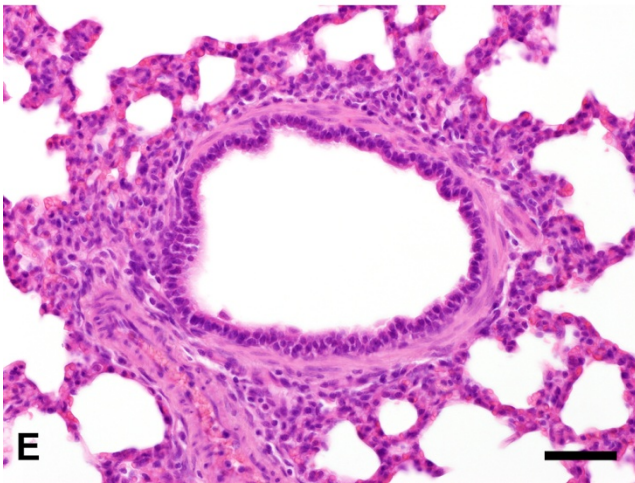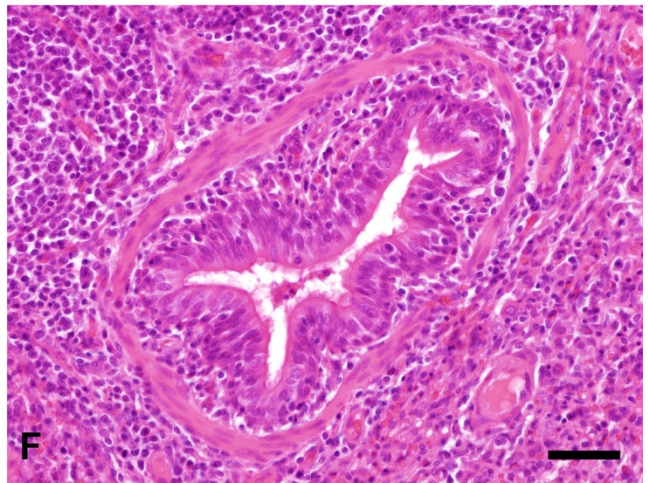

Supplement: Additional file 3: — Representative macroscopic and histologic lesions in a FLUAVsw-infected pig (#4) in comparison to a control animal (#2). The control animal shows no macroscopic alterations at the edges of the right cranial and middle lung lobes (A). Histologically, the lung tissue of the control animal shows thin alveolar walls and no inflammatory cells in the alveolar spaces and alveolar septa (C). The bronchioles are lined by a cuboidal epithelium and there are few mononuclear cells in the surrounding interstitium (E). In the infected animal there are several consolidated and retracted lobules at the ventral edge of the cranial and middle lobe (B). Microscopically, there is diffuse infiltration with mononuclear cells, fibrosis and metaplastic transformation of alveolar epithelia to cuboidal formations (D). Bronchiolar epithelia are markedly hyperplastic with pseudostratified columnar appearance and the surrounding interstitium is infiltrated with numerous mononuclear cells (F). Bars: 150 μm (C,D); 80 μm (E,F). [file 13567_2015_182_MOESM3_ESM.pdf]

A

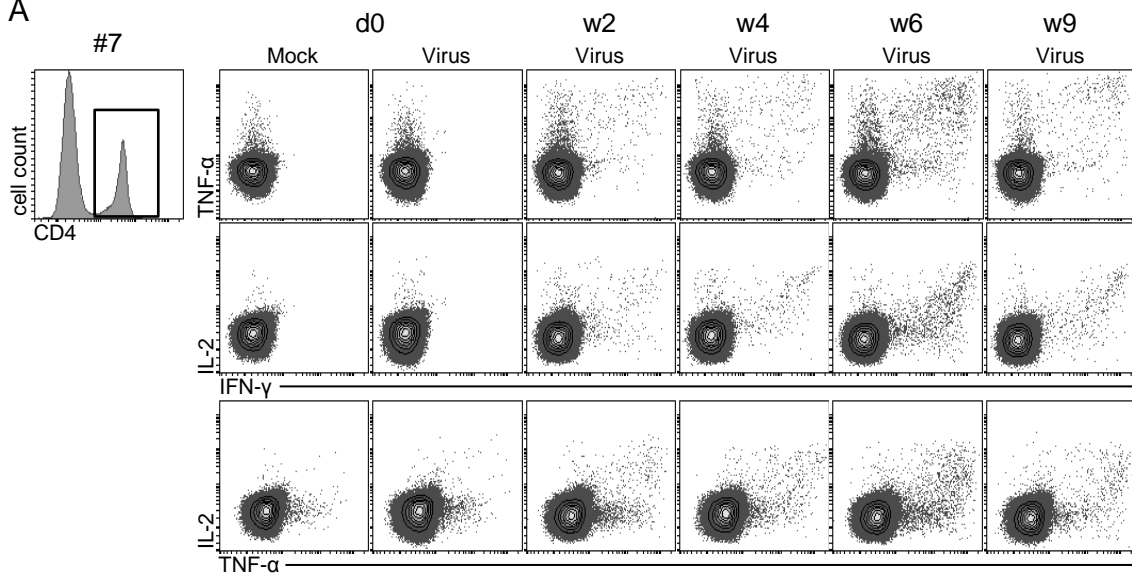

B

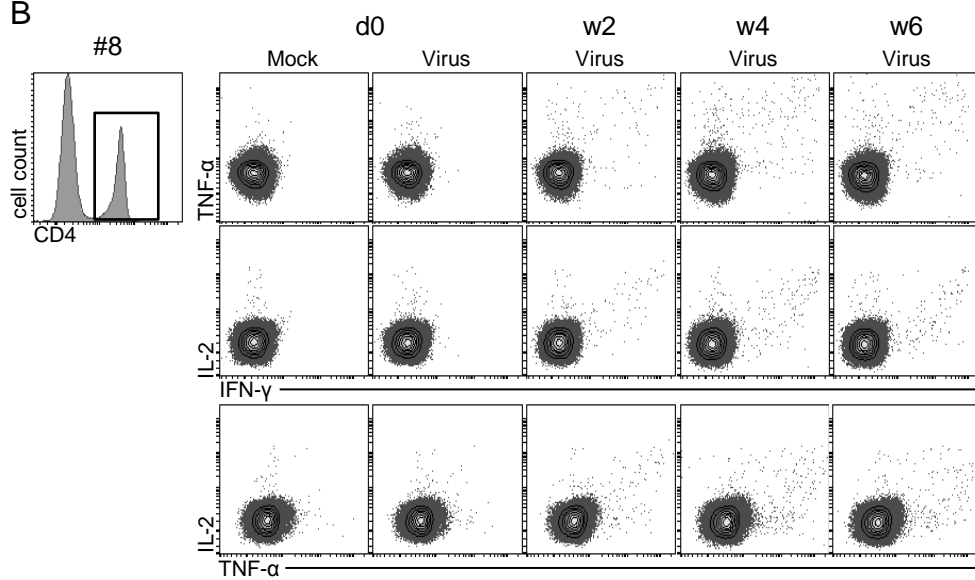

Supplement: Additional file 4: — Kinetics of FLUAVsw-specific IFN- γ -/TNF- α -/IL-2-producing CD4 + T cells. Intracellular cytokine staining of defrosted PBMCs was performed following overnight in vitro restimulation with FLUAVsw (infection strain, MOI = 0.1; 18 h). Mock-incubated cultures served as negative controls. CD4+ T cells were gated and analyzed for production of IFN-γ, TNF-α and IL-2. Contour plots show combinations of cytokines for selected time points following FLUAVsw infection. (A) Exemplary data of animal #7 (intermediate response) and (B) animal #8 (low response) is shown. [file 13567_2015_182_MOESM4_ESM.pdf]

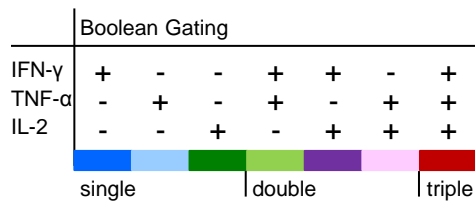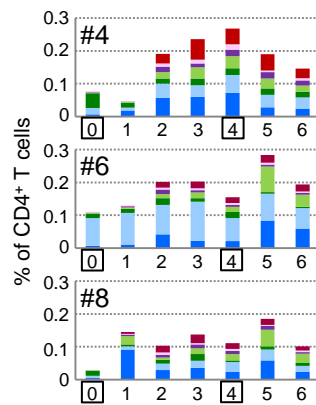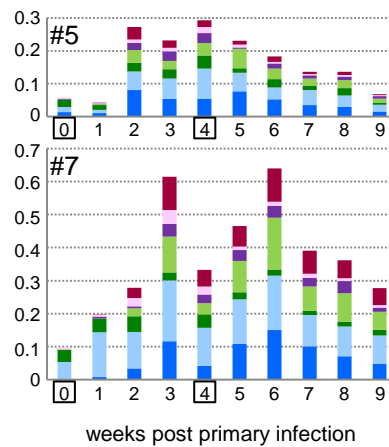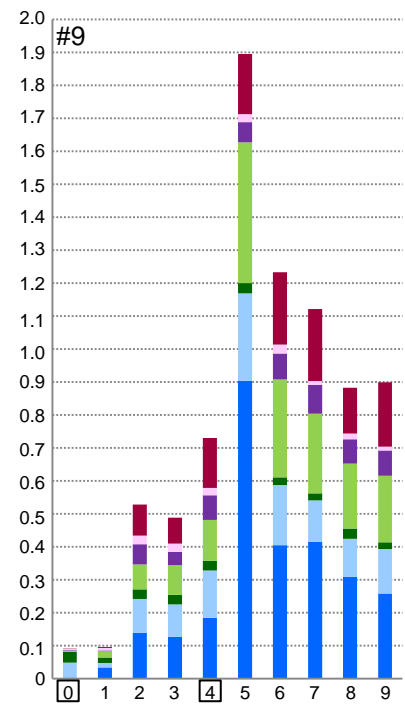

Supplement: Additional file 5: — Kinetics of FLUAVsw-specific CD4 + T-cell subsets producing different cytokines. Intracellular cytokine staining of defrosted PBMCs was performed following overnight in vitro restimulation with FLUAVsw (infection strain, MOI = 0.1; 18 h). Mock-incubated cultures served as negative controls. CD4+ T cells were gated and analyzed for production of IFN-γ, TNF-α and IL-2 as in Figure 4. Boolean gating was applied in order to identify all seven subsets of cytokine-producing CD4+ T cells. The frequency of the seven different cytokine-producing T-cell subsets are shown in stacked bar charts as percent of total CD4+ T cells for all six infected animals in the time course following FLUAVsw infection. [file 13567_2015_182_MOESM5_ESM.pdf]

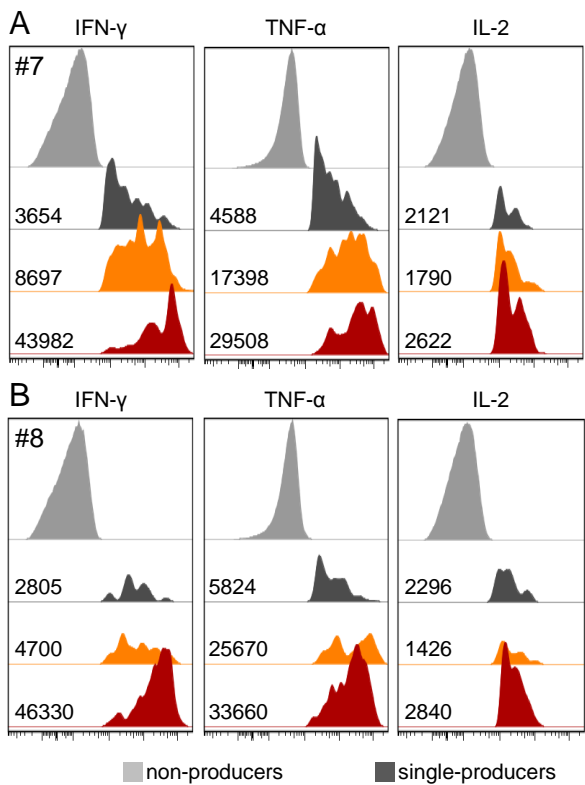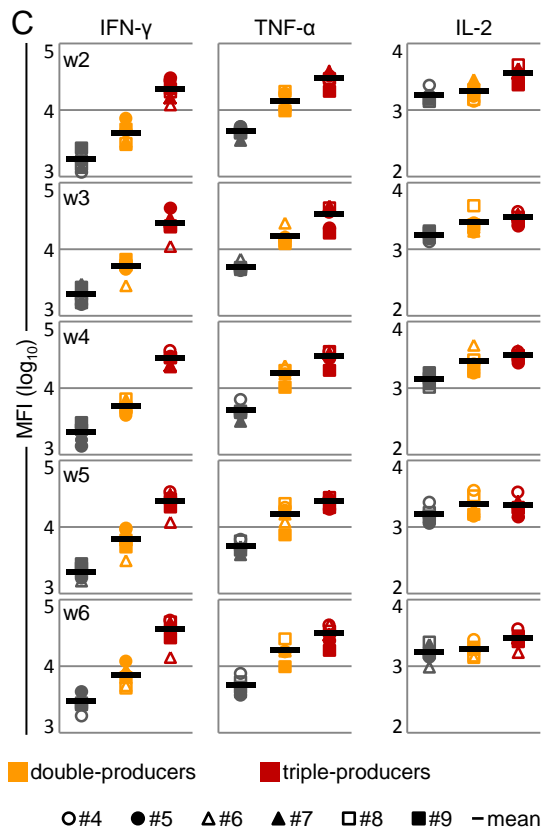

Supplement: Additional file 6: — IFN- γ/TNF- α/IL-2 expression levels of cytokine-producing CD4 + T cells. (A + B) Fluorescence intensity of IFN-γ, TNF-α and IL-2 expression within single- (dark grey), double- (orange) and triple- (red) cytokine-producing CD4+ T cells. Histograms show data of cells isolated from animal #7 (A; intermediate response) and animal #8 (B; low response) at six weeks post primary infection. Numbers indicate median fluorescence intensity (MFI) for each subset and the respective cytokine. (C) MFI of IFN-γ, TNF-α and IL-2 expression within single- (dark grey), double- (orange) and triple- (red) cytokine-producing CD4+ T cells isolated at two to six weeks post primary infection. Data of all six infected animals is shown by individual symbols. The mean is indicated by the black bar. [file 13567_2015_182_MOESM6_ESM.pdf]

A

#7

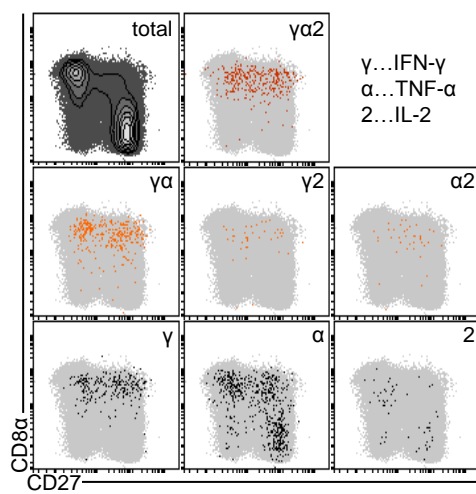

B

#8

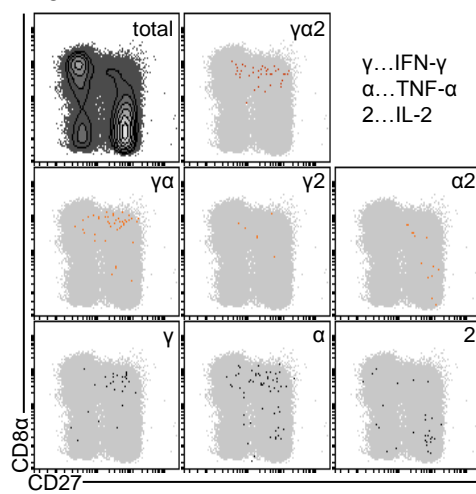

Supplement: Additional file 7: — CD8α /CD27 expression of cytokine-producing CD4 + T cells. CD8α and CD27 expression on total CD4+ T cells (contour plot in the upper left and light grey dots in dot plots) and CD4+ T cells producing a single cytokine (dark grey dots, bottom), two cytokines (orange dots, middle) or three cytokines (red dots, top). Data of CD4+ T cells from animal #7 (A; intermediate response) and animal #8 (B; low response) at six weeks post primary infection is shown. [file 13567_2015_182_MOESM7_ESM.pdf]

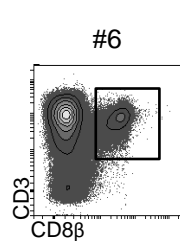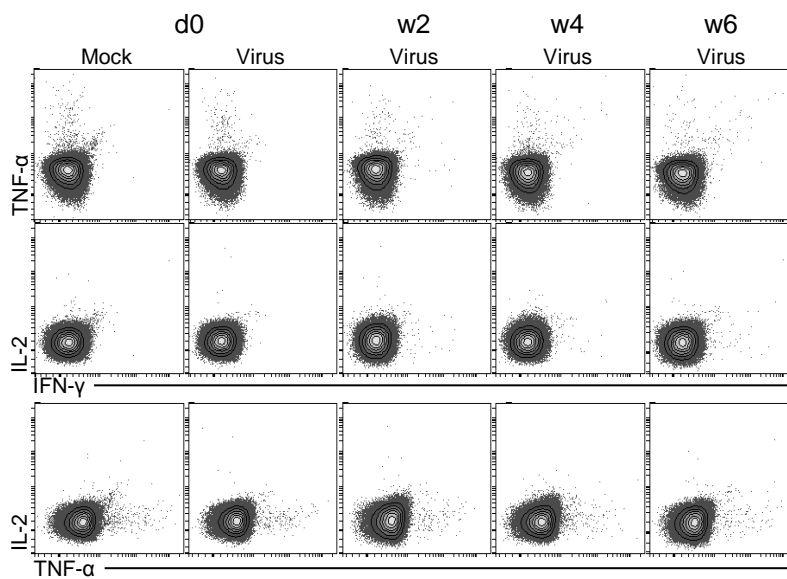

Supplement: Additional file 8: — Production of IFN-γ , TNF-α and IL-2 by FLUAVsw-specific CD8β + T cells. Intracellular cytokine staining of defrosted PBMCs was performed following overnight in vitro restimulation with FLUAVsw (infection strain, MOI = 0.1; 18 h). Mock-incubated cultures served as negative controls. CD3+CD8β+ T cells were gated and analyzed for production of IFN-γ, TNF-α and IL-2. Contour plots show combinations of cytokines for selected time points following FLUAVsw infection. Exemplary data obtained with CD8β+ T cells of animal #6 is shown. [file 13567_2015_182_MOESM8_ESM.pdf]
