# Supplementary material for: Magnitude and kinetics of multifunctional CD4+ and CD8β+ T cells in pigs infected with swine influenza A virus
Source: Vet Res. 2015 May 14;46(1):52. doi: 10.1186/s13567-015-0182-3 (PMC4429459; doi:10.1186/s13567-015-0182-3)
Supplement: Additional file 9: — Multifunctional FLUAVsw-specific CD8β + T cells identified by proliferation, CD107a expression and cytokine production. Violet-labeled PBMCs isolated at 2, 6 and 9 weeks post primary infection were restimulated twice with FLUAVsw (infection strain, MOI = 0.1; day 0 and day 5 of culture). On day 6 of culture, PBMCs were analyzed by FCM for proliferation, CD107a expression and production of IFN-γ and TNF-α. Boolean gating was applied in order to identify multifunctional subsets of CD8β+ T cells. All 11 multifunctional subsets (i.e. ≥ 2 functions) are shown in the left panel as stacked bar charts in % of total CD8β+ T cells. In the right panel, the subsets are grouped according to the number of functions they exert (as shown in Figure 6B). Data of six FLUAVsw-infected animals (#4-9) and two non-infected control animals (#1, #3) is shown. [file 13567_2015_182_MOESM9_ESM.pdf]

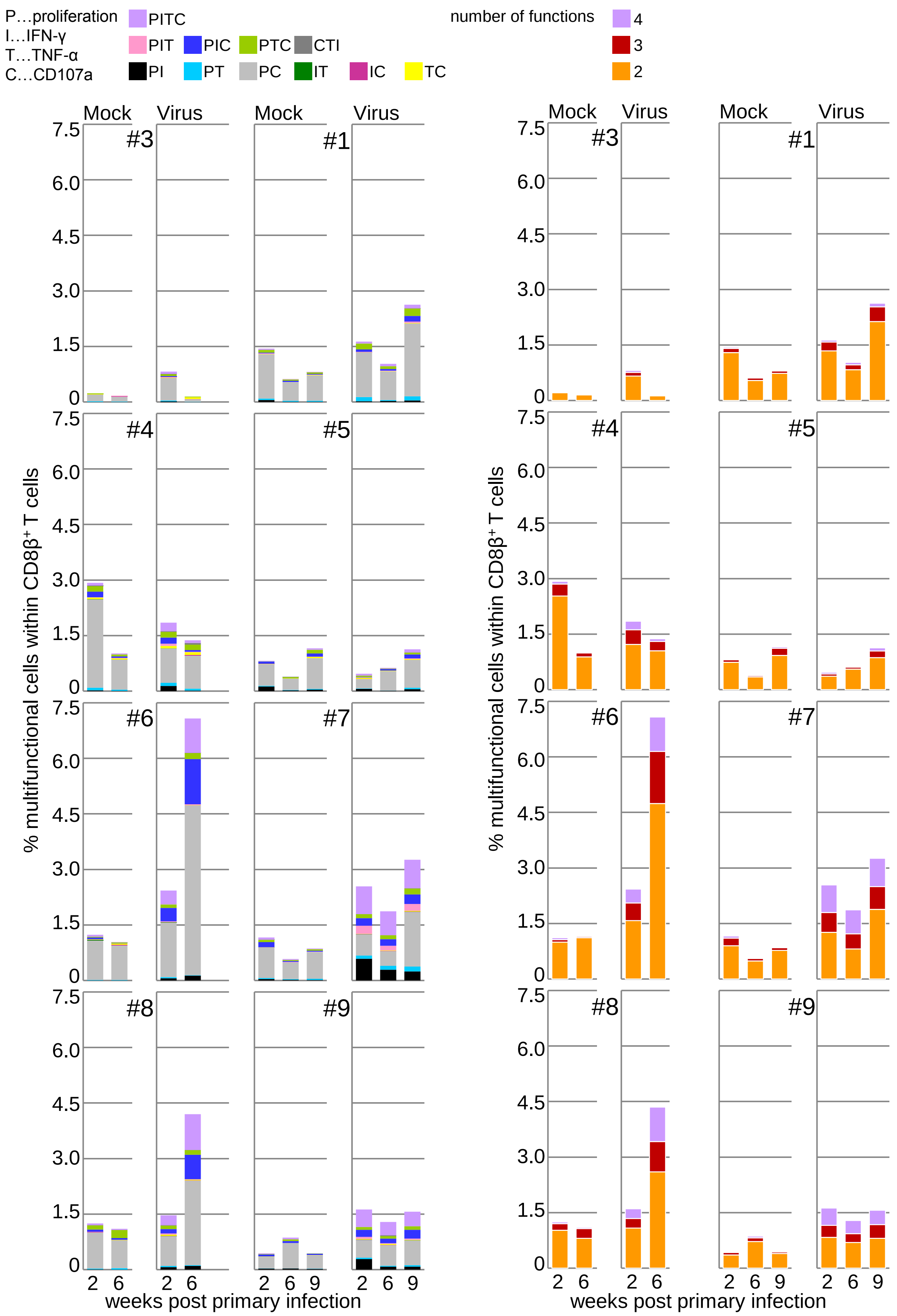

P…proliferation
I…IFN-γ
T…TNF-α
C…CD107a
PITC
PIT PIC PTC CTI
PI PT PC IT IC TC
number of functions
4
3
2
Mock Virus Mock Virus
#3 #1 #4 #5 #6 #7 #8 #9
% multifunctional cells within CD8β+ T cells
0 1.5 3.0 4.5 6.0 7.5
2 6 2 6 2 6 9 2 6 9
weeks post primary infection
